# Can HIV infection during pregnancy cause an intrauterine growth restriction?

**DOI:** 10.1186/1471-2334-13-S1-O5

**Published:** 2013-12-16

**Authors:** Simona Claudia Cambrea, Doina Eugenia Tănase, Maria Margareta Ilie, Simona Diaconu, Consuela Marcaş, Dalia Sorina Carp, Stela Halichidis, Lucian Cristian Petcu

**Affiliations:** 1 Clinical Infectious Diseases Hospital of Constanța, Romania; 2 Faculty of Medicine, “Ovidius” University, Constanța, Romania

## Background

Intrauterine growth restriction (IUGR) indicates the presence of a pathophysiological process occurring in utero that inhibits fetal growth. IUGR has been associated with increased perinatal and infant mortality. There are many maternal factors responsible for IUGR. Some studies have suggested that HIV infection could increase the risk of IUGR. Specifically, in IUGR, the baby's estimated weight is below the 10th percentile. The objective of this study was to evaluate the proportion of children born to HIV positive women who presented IUGR.

## Methods

We performed a retrospective study on the relevant parameters in newborns and mothers: demographic data, duration and type of combined antiretroviral treatment (cART). We analyzed anthropometric data of the newborn: weight, length, cranial circumference, and Apgar score. Statistical analysis was performed using MedCalc Software 2013.

## Results

Over a period of 5 years and 8 months, 124 newborn to 97 HIV+ mothers have been monitored. The median age in mothers was 22 (95%CI: 22-23), and mean 22.8 (range: 17 to 39). The mean Apgar score in newborns was 8.54 (range: 4 to 10), and median 9. The mean birth weight in newborns was 2670 g (range: 1000 to 3900), and median 2700 g. The proportion of children with birth weight less than 10th percentile was 58.05%. The mean length was 47.5 cm (range: 40 to 57), with a proportion of children below the 10th percentile of 29.26%. Infants who presented below the 10th percentile for weight and length were 22.76%. About 11.38% of infants were below the 10th percentile for weight, length and cranial circumference. 97.5% of children received cART after birth, and 93.1% of mothers received cART during pregnancy. In the studied period the mortality rate was 5.6% in children and 7.2% in mothers.

## Conclusion

More than half (58.53%) of the infants born to HIV positive mothers were small for gestational age. 22.76% of infants were with IUGR, and 11.38% of them presented symmetrical IUGR.

